# Centromere Identity and the Regulation of Chromosome Segregation

**DOI:** 10.3389/fcell.2022.914249

**Published:** 2022-06-02

**Authors:** Kousik Sundararajan, Aaron F. Straight

**Affiliations:** Department of Biochemistry, Stanford University School of Medicine, Stanford, CA, United States

**Keywords:** centromere, CENP-A, chromatin, epigenetics, alpha satellite

## Abstract

Eukaryotes segregate their chromosomes during mitosis and meiosis by attaching chromosomes to the microtubules of the spindle so that they can be distributed into daughter cells. The complexity of centromeres ranges from the point centromeres of yeast that attach to a single microtubule to the more complex regional centromeres found in many metazoans or holocentric centromeres of some nematodes, arthropods and plants, that bind to dozens of microtubules per kinetochore. In vertebrates, the centromere is defined by a centromere specific histone variant termed Centromere Protein A (CENP-A) that replaces histone H3 in a subset of centromeric nucleosomes. These CENP-A nucleosomes are distributed on long stretches of highly repetitive DNA and interspersed with histone H3 containing nucleosomes. The mechanisms by which cells control the number and position of CENP-A nucleosomes is unknown but likely important for the organization of centromeric chromatin in mitosis so that the kinetochore is properly oriented for microtubule capture. CENP-A chromatin is epigenetically determined thus cells must correct errors in CENP-A organization to prevent centromere dysfunction and chromosome loss. Recent improvements in sequencing complex centromeres have paved the way for defining the organization of CENP-A nucleosomes in centromeres. Here we discuss the importance and challenges in understanding CENP-A organization and highlight new discoveries and advances enabled by recent improvements in the human genome assembly.

## Centromeres are Epigenetically Defined

Centromeres provide the site on each chromosome where spindle microtubules attach during cell division. The multiprotein complex known as the kinetochore assembles at the centromere and attaches to dynamic microtubules generating the primary binding interface between the chromosome and mitotic spindle fibers. The kinetochore nucleoprotein complex is necessary for chromosome orientation during metaphase, mitotic checkpoint activity, and segregation of daughter chromosomes during anaphase (Cleveland et al., 2003; Musacchio and Desai, 2017).

Centromeres vary widely in their organization and structure between organisms but one shared function is their activity in generating a microtubule binding site. Chromatin in which the histone H3 variant, CEntromere Protein A (CENP-A), replaces histone H3 in a subset of centromeric nucleosomes determines the position of the active centromere and kinetochore (Westhorpe and Straight, 2014). Budding yeast, for example, has a point centromere where DNA wraps a single CENP-ACse4 nucleosome to define the active centromere region (Furuyama and Biggins, 2007). In fission yeast and many metazoans, “regional centromeres” are assembled on repetitive DNA sequences and flanked by domains of heterochromatin (Pidoux and Allshire, 2005; Allshire and Karpen, 2008). However, the active centromere is generally a smaller region within the centromeric repeat and pericentromeric heterochromatin domain (Warburton et al., 1997). Within regional centromeres, CENP-A nucleosomes are interspersed with H3 nucleosomes and sparsely distributed (Van Hooser et al., 2001; Blower et al., 2002; Sullivan and Karpen, 2004). Although repeat DNA can span megabases in many organisms CENP-A occupies a small subdomain of the entire centromere and pericentromeric repeats (Blower et al., 2002; Spence et al., 2002; Zeng et al., 2004; Lam et al., 2006; Sullivan et al., 2011). How cells determine and maintain the position of CENP-A (and therefore, the site of microtubule attachment) within the centromere is a key question in centromere biology. The answers to these questions are within our reach now thanks to new experimental methods and the completion of the human genome assembly that includes high confidence and contiguous centromere sequences (Altemose et al., 2022a, 2022b; Gershman et al., 2022; Hoyt et al., 2022; Nurk et al., 2022).

### Organizing CENP-A Nucleosomes Within the Centromere

Electron micrographs of metaphase arrested human cells suggest that there are on average ∼20 kinetochore/microtubules attached at each centromere (McEwen et al., 2001). Within the centromere of the mitotic chromosome, CENP-A nucleosomes are organized in three dimensions on the surface of chromosome, possibly to promote assembly of the kinetochore for efficient microtubule capture and attachment (Marshall et al., 2008b). More recently, super resolution microscopy of CENP-A in human U2OS cells has revealed a dense rosette-like organization of CENP-A nucleosomes in G1 immediately following HJURP-mediated deposition and rod-like elongated organization in mitosis (Andronov et al., 2019). Studies of centromere organization using microscopic techniques have proposed several structures for the three dimensional organization of the centromere (Blower et al., 2002; Marshall et al., 2008b; Ribeiro et al., 2010). However, the relationship between the one-dimensional position of CENP-A within centromere DNA sequences and the three-dimensional organization that forms a functional centromere is unknown.

The two essential centromere proteins that recognize and directly bind CENP-A nucleosomes—CENP-C and CENP-N—can both bind more than one nucleosome (Carroll et al., 2009, 2010; Kato et al., 2013; Zhou et al., 2022). Both CENP-C and CENP-N have been proposed to cluster CENP-A nucleosomes within the centromere. CENP-C is a dimer and has two nucleosome binding motifs that interact with CENP-A by binding the CENP-A C-terminus and the H2A/H2B acidic patch (Sugimoto et al., 1997; Cohen et al., 2008; Carroll et al., 2010; Kato et al., 2013; Walstein et al., 2021). CENP-C depletion makes CENP-A chromatin more susceptible to salt-dependent extension of centromere chromatin (Ribeiro et al., 2010). CENP-N has two binding sites for the nucleosome, one that is specific to CENP-A through interaction with the RG-loop and a second binding site that interacts with the DNA and does not distinguish between the nucleosome type (Pentakota et al., 2017; Chittori et al., 2018; Zhou et al., 2022). These two activities have been shown to bridge CENP-A nucleosomes or link CENP-A and H3 nucleosomes (Zhou et al., 2022). In cells, CENP-N promotes the compaction of centromeric chromatin as assayed by sedimentation of chromatin in sucrose gradients after sonication. Together, CENP-C and CENP-N are able to link centromeric nucleosomes together and are important for the compaction and organization of centromeric chromatin in cells.

**FIGURE 1:**
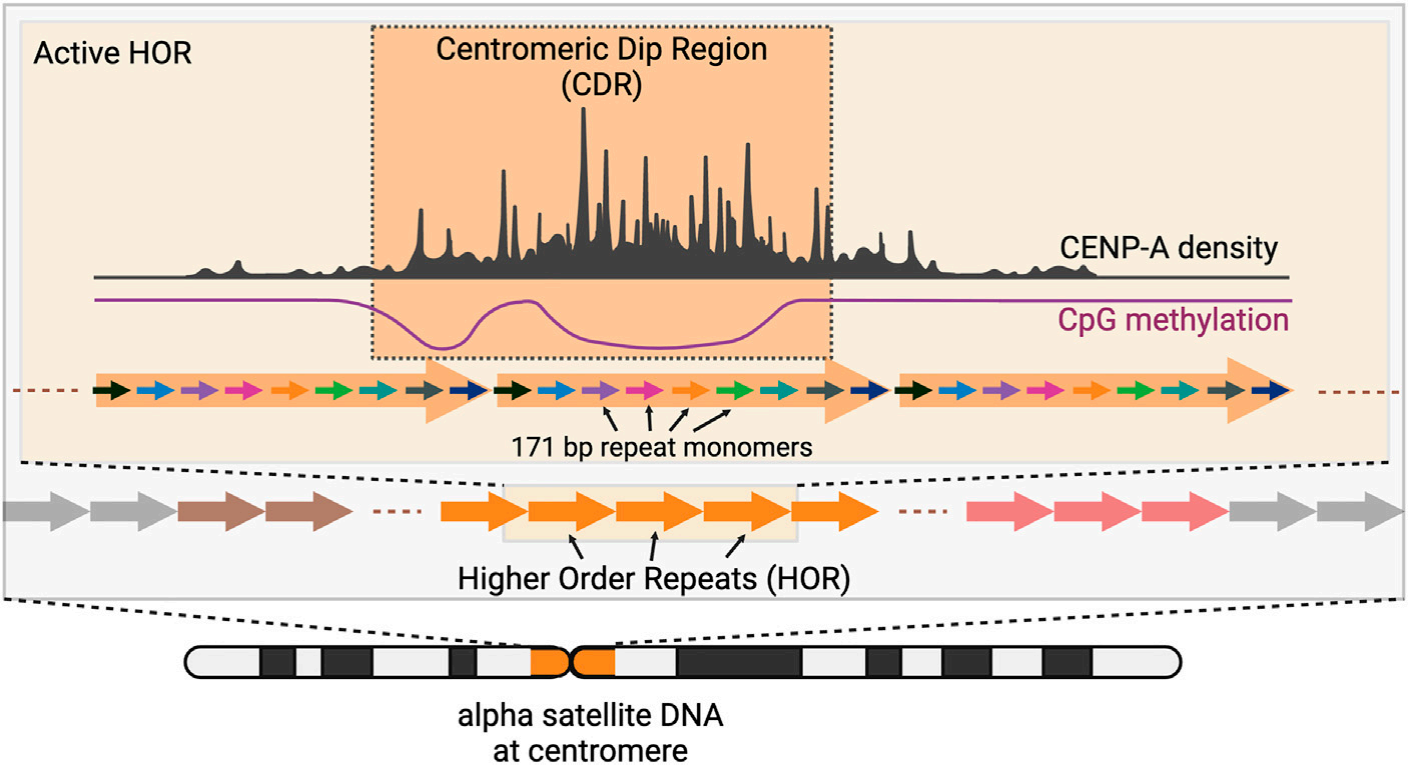
Centromeres contain arrays of higher order repeats (HORs) made of ∼171 bp alpha satellite repeat monomers. CENP-A nucleosomes are organized within the “active” HOR of the chromosome and are found enriched at the region of low local CpG methylation also known as Centromeric Dip Region or CDR. (Created with BioRender.com).

Centromere and pericentromere DNA make up ∼6% (189 Mb) of the genome (Altemose et al., 2022a; Nurk et al., 2022). Centromere DNA consists of highly repetitive DNA sequences known as alpha satellites (Figure 1) (Willard, 1985; Greig et al., 1989; Schueler et al., 2001). CENP-A nucleosomes are typically located within alpha satellite DNA which contain highly homogenous repeats of monomeric DNA repeats. These repeats, known as Higher Order Repeats (HOR), contain arrays of monomers of ∼171 bp arranged in tandem, and span between 340 kb (Chromosome 21) to 4.8 Mb (Chromosome 18) (Manuelidis and Wu, 1978; Willard, 1985; Waye and Willard, 1987; Warburton and Willard, 1990; Alexandrov et al., 1993; McNulty and Sullivan, 2018; Altemose et al., 2022a).

Chromatin fiber stretching experiments in human cells show dense clusters of CENP-A occupying approximately 30–40% of alpha satellite DNA on individual centromere repeat arrays, suggesting that CENP-A levels are proportional to the amount of centromere DNA on each chromosome (Sullivan et al., 2011). Chromatin fiber stretching is effective at probing the localization of histones and histone variants in chromatin but lacks the ability to quantify the exact number of nucleosomes within a region on a given chromosome. Moreover, the reliance on fluorescence *in situ* hybridization to identify centromere DNA sequence limits distinction between the highly similar repeat sequences at centromeres. Enrichment of CENP-A within a smaller portion of the active centromere has also been observed on neocentromeres i.e. *de novo* centromeres that form outside the canonical centromere (Marshall et al., 2008a; Naughton and Gilbert, 2020). In the naturally occurring neocentromere on chromosome 10 (mardel10), about 330 kb of a 3.5 Mb stretch of neocentromere region has a high density of CENP-A by ChIP and PCR-array analyses (Chueh et al., 2005). Within these 330 kb, there appear to be several contiguous regions of high CENP-A density (with one >50 kb and a few flanking regions ∼10–30 kb). Similarly, isolation of neocentromere-containing clones derived from chicken DT40 cells revealed a typical core subdomain of ∼40 kb on average within neocentromeres (Shang et al., 2013).

Fluorescence imaging in cells using endogenous fluorescent fusions to CENP-A have enabled more absolute quantification of centromere nucleosomes. In RPE1 cells the average human centromere contains ∼400 CENP-A molecules or 200 CENP-A nucleosomes (Bodor et al., 2014). These estimates assume an active centromere size of 2.5 Mb, 40% of which (i.e. 1 Mbp) corresponds to the CENP-A-enriched region (Sullivan et al., 2011). Individual centromeres within the same cell seem to vary up to 10-fold in their CENP-A levels but in the absence of directly labeling individual chromosomes, it is unclear from this study if these variations correspond to chromosome-specific differences (Sullivan et al., 2011). These estimates also vary widely across cell types, with DLD-1 cells containing as few as ∼90 CENP-A nucleosomes per centromere on average (Bodor et al., 2014).

The recent development of long read sequencing methods to confidently assemble alpha satellite DNA sequences allow investigations of CENP-A position by genomic techniques. Efforts to map centromeric nucleosomes using either CENP-A ChIP-seq or chromatin fiber spreading combined with chromosome 8 specific FISH show that CENP-A nucleosomes are clustered within a small portion of the alpha satellite DNA (Logsdon et al., 2021). Dense clustering of CENP-A nucleosomes within a subdomain of alpha satellite DNA has been observed on other chromosomes as well (Altemose et al., 2022a, 2022b; Gershman et al., 2022; Nurk et al., 2022). Because CENP-A nucleosomes reside on only a small fraction of the HOR sequences, there does not appear to be a strict relationship between CENP-A levels at a given centromere and the size of that centromere’s HOR. The active HOR region of the centromere where CENP-A is localized is usually found in the newer/younger regions of the centromere as assessed by the degree of divergence of the HOR monomer sequence and the absence of conserved insertions/deletions compared to other primates (Alexandrov et al., 2001; Logsdon et al., 2021; Altemose et al., 2022a). This supports the idea that centromere sequences evolve rapidly and that CENP-A position and centromere function drive this evolution.

The active HOR can vary within the population and sometimes even within the individual. A well studied example is the Chromosome 17 centromere. Two epialleles of the active HOR have been identified (Rudd et al., 2006; Maloney et al., 2012; Aldrup-MacDonald et al., 2016) with one epiallele being more common in the population. The recent assembly of the chromosome X centromere has confirmed the presence of additional epialleles (Miga et al., 2020; Altemose et al., 2022a). For example, CENP-A nucleosomes are differentially positioned on an older HOR in a subpopulation of cells and the two X chromosomes of RPE1 cells have CENP-A at different positions. These observations suggest that CENP-A position can vary within a population. The physiological consequence of CENP-A placement on these different epialleles is unclear.

Curiously, CENP-A nucleosome bound DNA is associated with regions of cytosine hypomethylation of CpG sequences (Logsdon et al., 2021; Gershman et al., 2022). Centromeres are generally hypermethylated, however, a region within each centromere is hypomethylated compared to its neighbors, dubbed the “Centromere Dip Region” or CDR (Gershman et al., 2022). The HOR containing the CDR is enriched for CENP-A ChIP-seq reads suggesting a correlation between DNA methylation levels and the site of CENP-A assembly. Even at the level of individual chromatin fibers (representing individual chromosomes), CENP-A nucleosomes are ∼5 fold enriched within a 100 kb region spanning the CDR compared to adjacent regions (Altemose et al., 2022b). The likelihood of a CENP-A nucleosome increases from 1 in 25 across the HOR (on average) (Bodor et al., 2014) to 1 in 4 within the CDR (Altemose et al., 2022b).

### Homeostatic Control of CENP-A Chromatin

Like histone H3 nucleosomes, CENP-A nucleosomes are distributed stochastically to daughter chromosomes in roughly equal amounts during DNA replication (Shelby et al., 2000). However, unlike histone H3 nucleosomes, new CENP-A nucleosomes are not deposited immediately following replication fork progression. Vertebrate cells and *Caenorhabditis elegans*, assemble CENP-A nucleosomes after cells exit mitosis in the following G1 (Jansen et al., 2007; Maddox et al., 2007; Bernad et al., 2011; Moree et al., 2011; Silva et al., 2012). In *Drosophila melanogaster*, somatic cells load new CENP-A into chromatin in metaphase (Mellone et al., 2011) while early embryos assemble CENP-A in early anaphase (Schuh et al., 2007). *Schizosaccharomyces pombe* and *Arabidopsis thaliana* both form new CENP-A nucleosomes immediately after replication in G2 (Lermontova et al., 2006; Takayama et al., 2008; Lando et al., 2012). In all of these systems, CENP-A nucleosome assembly is uncoupled from replication but serves to restore CENP-A levels after dilution during replication.

The uncoupling of the CENP-A assembly process from the distribution of CENP-A nucleosomes during replication poses a challenge to the cells for maintaining CENP-A within centromeric chromatin. During replication, daughter chromosomes inherit CENP-A nucleosomes as homotypic, octameric nucleosomes (Hasson et al., 2013; Padeganeh et al., 2013; Nechemia-Arbely et al., 2017). Each sister centromere inherits approximately 50% (+/− 12%) of CENP-A on average, however, each CENP-A nucleosome is seemingly randomly distributed to either sister centromere (Bodor et al., 2014; Ross et al., 2016). Such stochastic distribution suggests that the position and number of CENP-A nucleosomes inherited at each centromere could vary significantly from generation to generation unless the cell compensates for unequal centromeric nucleosome distribution. ChIP-seq of CENP-A bound sequences before and after replication in cell cycle synchronized cells shows that CENP-A is enriched on the same centromeric sequences before and after replication (Nechemia-Arbely et al., 2019) supporting that inherited CENP-A nucleosomes are still retained at the same positions overall irrespective of the daughter chromosome.

**FIGURE 2:**
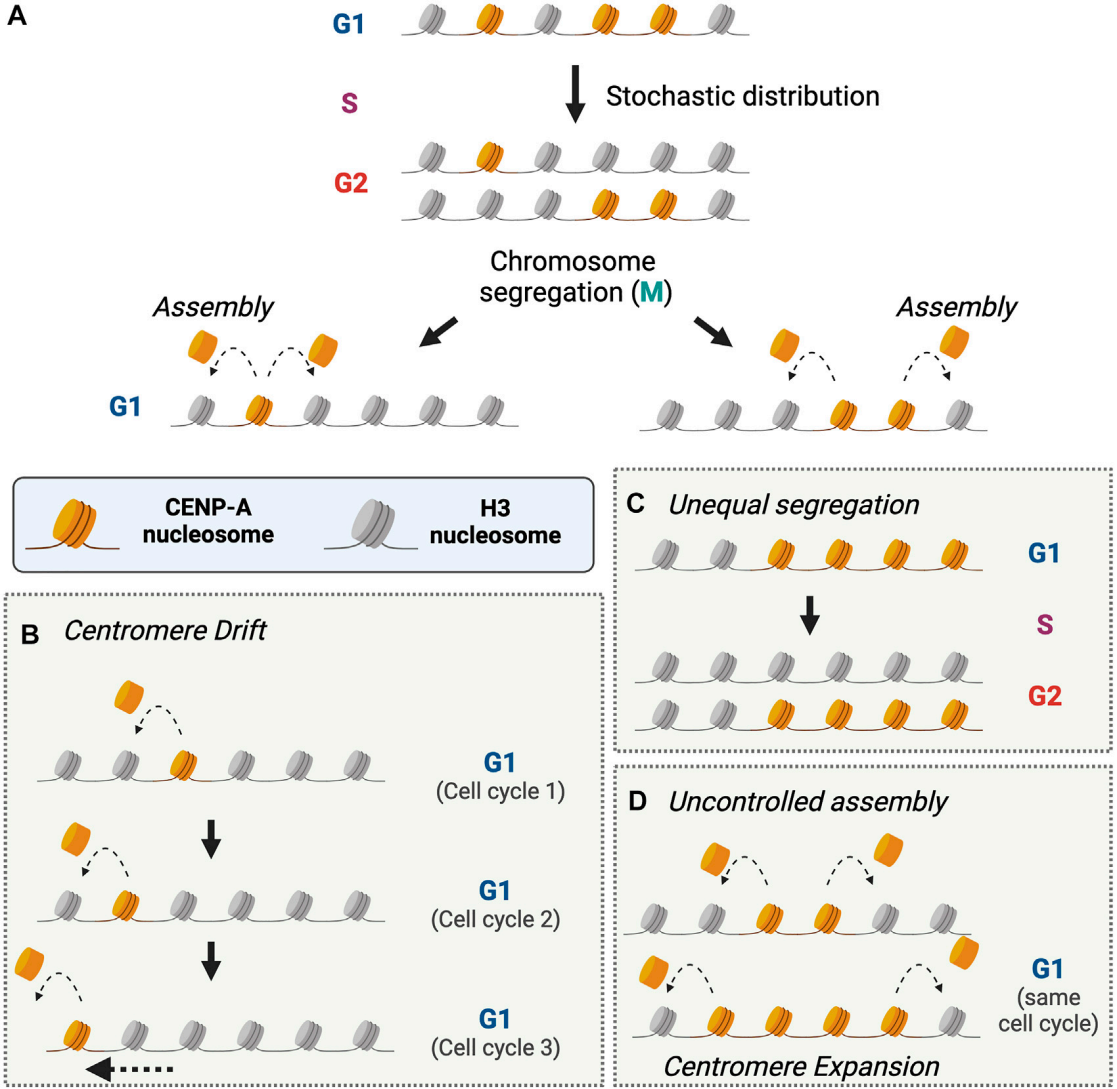
Regulation of CENP-A segregation and assembly to maintain centromere identity. **(A)**. CENP-A nucleosomes are stochastically distributed to newly replicated chromosomes during DNA replication. During the next G1-phase, CENP-A nucleosomes that were distributed during replication promote the deposition of new CENP-A nucleosomes to replenish CENP-A at centromeres. Unregulated CENP-A deposition can result in loss of centromere identity within a few cell cycles as described in **(B–D)**. How cells prevent centromere drift, loss, or expansion is not explained by a CENP-A-templated assembly model. **(B)**. Assembly of CENP-A nucleosomes has the potential to allow drift of the centromere over multiple rounds of replication and assembly. **(C)**. Centromere expansion can occur due either to unequal segregation during replication or **(D)**. if multiple rounds of CENP-A nucleosome assembly can be directed by a single CENP-A nucleosome. (Created with BioRender.com).

CENP-A nucleosome assembly is epigenetically influenced such that the assembly of new CENP-A depends on the presence of preexisting CENP-A nucleosomes. On average, CENP-A nucleosome assembly restores the amount of CENP-A in chromatin after replication by doubling the number of nucleosomes. Biochemical analysis of CENP-A assembly factors has provided some mechanistic insights into this process. A predominant model (Nardi et al., 2016; Pan et al., 2017, 2019) is that a CENP-A nucleosome templates the assembly of a new CENP-A nucleosome in order to regenerate the pre-replicative levels of CENP-A in chromatin (Figure 2A). CENP-A nucleosomes appear to be deposited proximal to the existing CENP-A nucleosomes (Dunleavy et al., 2011; Ross et al., 2016; Shukla et al., 2018). If a new CENP-A nucleosome can form on either side of a preexisting CENP-A nucleosome, or on a non-adjacent site away from the active centromere, this would result in drift of the location of CENP-A chromatin and dilution of CENP-A density within a few generations unless other mechanisms constrain the location of the centromere (Figure 2B).

Neocentromeres are thought to arise on chromosome fragments or rearranged chromosomes where the canonical centromere is no longer functional (Barra and Fachinetti, 2018). The mechanism of neocentromere biogenesis is unclear, but it is possible that mistakes in CENP-A maintenance within centromeres could result in neocentromere formation. Observations of neocentromeres on chromosomes without obvious rearrangements (Tyler-Smith et al., 1999; Amor et al., 2004; Ventura et al., 2004; Liehr et al., 2010) support that neocentromeres can also result from expansion and/or drift of centromeric chromatin (Naughton and Gilbert, 2020).

Centromere drift has been observed in nonrepetitive centromeres where the position of CENP-A nucleosomes can be unambiguously mapped. Studies in chicken DT40 cells showed that the nonrepetitive Z centromere varied in its position from cell to cell indicating movement of the centromere during development. However, when individual cells were cloned the centromere maintained a stable position over 50 generations. Compromising centromere function with nonlethal mutations in centromere proteins caused an increase in the rate of centromere movement indicating that the proteins of the centromere may constrain the position of CENP-A nucleosomes (Hori et al., 2017a). A similar study examining horse chromosome 11 demonstrated changes in centromere position between 5 individuals suggesting that centromere position fluctuates across the population (Purgato et al., 2015). These studies take advantage of non-repetitive centromeres to unambiguously map CENP-A positions to investigate centromere identity across the population and through generations. Mapping CENP-A nucleosomes confidently on repetitive centromere sequences will be instrumental for similar investigations of the maintenance of centromere identity in other metazoan centromeres including humans.

A prediction of a CENP-A templated assembly is that centromeres with fewer CENP-A nucleosomes (resulting from stochastic distribution during replication) will receive fewer CENP-A nucleosomes in the next cell cycle, essentially doubling disparities in CENP-A levels between daughter cells. Tracking CENP-A deposition using pulse-labeling of exogenous SNAP-CENP-A shows poor correlation in the extent of asymmetric distribution of CENP-A nucleosomes to sister centromeres between consecutive cell cycles, suggesting that replicative distribution is not regulated to correct for low abundance of CENP-A nucleosomes (Ross et al., 2016). Therefore, extreme disparities in CENP-A inheritance amongst daughter chromosomes, however rare, needs to be compensated for during CENP-A assembly (Figure 2C). Interestingly, CENP-A deposition appears to be driven by mass action; within a 5-fold range of artificial manipulations in CENP-A expression, CENP-A density within centromeres varies proportional to the total cellular CENP-A levels (Bodor et al., 2014). Furthermore, CENP-A levels at centromeres are rapidly restored to physiological levels within 2 cell cycles after transient CENP-A depletion through targeted degradation (Hoffmann et al., 2020). These observations suggest that there may be compensatory mechanisms for restoring normal CENP-A nucleosome levels other than strictly doubling the number of pre-existing CENP-A nucleosomes at each centromere.

### Epigenetic Mechanisms of CENP-A Chromatin Maintenance

In humans and other vertebrates, the histone chaperone HJURP binds a CENP-A:H4 dimer prior to CENP-A nucleosome assembly (Dunleavy et al., 2009; Foltz et al., 2009). Early in G1-phase, HJURP is recruited to centromeres by binding CENP-C and the Mis18 complex to deliver new CENP-A to centromeric chromatin (Carroll et al., 2010; Barnhart et al., 2011; Moree et al., 2011; Kato et al., 2013; Kral, 2015; Tachiwana et al., 2015; Nardi et al., 2016; Hori et al., 2017b; French et al., 2017; Pan et al., 2017, 2019; Sandmann et al., 2017; Walstein et al., 2021). HJURP localization to chromatin is sufficient for CENP-A deposition - ectopic localization of LacI-HJURP to a non-centromeric LacO array in human U2OS cells is able to deposit CENP-A on a chromosome arm to form functional kinetochores (Barnhart et al., 2011). Similarly, ectopic localization of CENP-C using the LacI/LacO system is sufficient for ectopic CENP-A deposition, presumably through the recruitment of HJURP (Gascoigne et al., 2011). Thus, directly or indirectly targeting HJURP to chromatin appears to be sufficient for deposition of CENP-A nucleosomes.

A key unanswered question is how does the cell control the number of CENP-A nucleosomes assembled or maintained in chromatin to ensure that the centromere is regenerated after dilution by replication. One proposal is that the human Mis18 complex that binds to the centromere during G1 may license the assembly of CENP-A in a manner analogous to DNA replication licensing (Fujita et al., 2007). The cell cycle dependent targeting of the Mis18 complex, followed by dissociation of the complex upon HJURP binding to prevent uncontrolled assembly of CENP-A nucleosomes has been proposed to prevent over-expansion of CENP-A chromatin (Nardi et al., 2016). However, recent evidence indicates that the Mis18 complex remains stable upon HJURP binding, bringing this licensing model into question (Pan et al., 2019). An alternate model based on recent biochemical reconstitution studies proposes that CENP-C recognizes adjacent CENP-A and H3 dinucleosomes and promotes HJURP recruitment and CENP-A deposition to replace the H3 nucleosome (Pan et al., 2019; Walstein et al., 2021). In this model, a CENP-A:H3 dinucleosome templates the assembly of one CENP-A nucleosome. However, it is unclear what would prevent an additional round of CENP-A nucleosome assembly if CENP-A were positioned between two H3 nucleosomes (Figure 2D). While one could imagine that CENP-A deposition continues until exhaustion of all CENP-A:H3 dinucleosomes within centromeres, the low density of CENP-A within centromeres in cells prior to replication suggests otherwise (Bodor et al., 2014; Nechemia-Arbely et al., 2019). Only 20% of cellular CENP-A is deposited within centromeres, further suggesting that CENP-A assembly process is not limited by levels of soluble CENP-A alone (Bodor et al., 2014). CENP-C, Mis18 complex, HJURP, or another factor could distinguish between a pre-existing CENP-A and a newly deposited CENP-A, limiting uncontrolled deposition of CENP-A nucleosomes. The temporal regulation of CENP-C and/or Mis18 complex recruitment to centromeres prior to CENP-A assembly in early G1 could explain how cells make this distinction (Fujita et al., 2007; Maddox et al., 2007; Lagana et al., 2010; Silva et al., 2012; McKinley and Cheeseman, 2014; Wang et al., 2014; Nardi et al., 2016; Spiller et al., 2017; Stankovic et al., 2017; French and Straight, 2019). These factors could detect the presence or absence of modifications either on H3 or CENP-A of the dinucleosome to distinguish between pre-existing (inherited) and *de novo* (i.e. resulting from new CENP-A deposition) dinucleosomes to limit excessive deposition. Evidence for cell cycle regulated epigenetic modifications on CENP-A and centromeric H3 have been reported (for example (Bui et al., 2017; Niikura et al., 2019; Mitra et al., 2020), also reviewed in (Srivastava and Foltz, 2018)) but the extent of their influence on CENP-A deposition remains to be determined. While every CENP-A nucleosome may not be “licensed” to direct deposition of a single new CENP-A nucleosome, whether HJURP recruitment and new CENP-A deposition are restricted to assemble CENP-A nucleosomes proportional to existing CENP-A nucleosomes or to supplement missing CENP-A nucleosomes is unclear.

CENP-A nucleosomes can be deposited outside centromeres on chromosome arms in G1, but cells clear these ectopic nucleosomes from non-centromere DNA during DNA replication (Nechemia-Arbely et al., 2019). CENP-A redeposition during replication is mediated through HJURP (Zasadzińska et al., 2018) and requires CENP-C which remains at the centromere (Nechemia-Arbely et al., 2019). How cells distinguish centromeric CENP-A nucleosomes from ectopic ones is unclear but centromeric localization of CENP-C could selectively promote retention of CENP-A nucleosomes at the centromere and eviction of ectopic CENP-A nucleosomes. Proximity labeling of CENP-C and CENP-A on chromatin spreads suggest that both CENP-C and CENP-A are restricted to the active centromeres and they are present at similar densities (Kyriacou and Heun, 2018). Not all CENP-C loci contain CENP-A suggesting that CENP-C is tethered to centromeres through additional means. Furthermore, cells tolerate transient CENP-A depletion for one cell cycle, can undergo mitosis, and redeposit physiological levels of CENP-A in the next cell cycle in a CENP-C dependent manner (Hoffmann et al., 2016, 2020). Taken together, these observations suggest that cells co-regulate CENP-A and CENP-C, and they potentially depend on CENP-C for restoring physiological levels of centromeric CENP-A upon loss of CENP-A from chromatin.

### Genetic Determinants of CENP-A Location

While centromere sequences vary across chromosomes and between HORs, a common feature of all centromeres except chromosome Y in humans is the presence of a CENP-B box (Earnshaw et al., 1989; Masumoto et al., 1989; Muro et al., 1992; Yoda et al., 1992; Haaf and Ward, 1994; Miga et al., 2014). The CENP-B box, a 17 bp motif, binds the protein CENP-B (Earnshaw and Rothfield, 1985; Masumoto et al., 1989; Yoda et al., 1992). CENP-B can interact with CENP-A and CENP-C (Suzuki et al., 2004; Fachinetti et al., 2015). CENP-B is not required for CENP-A assembly (Fachinetti et al., 2015). However, after transient experimental depletion of CENP-A nucleosomes using artificial degron systems, cells assemble and restore CENP-A nucleosomes at centromeres in the next G1-phase in a CENP-B dependent manner (Hoffmann et al., 2020). CENP-B is not essential for centromere function or identity (Kapoor et al., 1998). Moreover, chromosome Y and neocentromeres do not have CENP-B boxes. However, centromeres lacking CENP-B boxes are more vulnerable to centromere dysfunction and chromosome segregation defects (Fachinetti et al., 2015; Hoffmann et al., 2020). Methylation of CpG’s within CENP-B boxes prevents CENP-B binding (Tanaka et al., 2005) suggesting that the methylation state of CENP-B boxes might locally control CENP-A deposition and/or maintenance. The CENP-A enrichment observed at CDR’s could be explained by the hypomethylation allowing more CENP-B binding and therefore more CENP-A recruitment at these sites. If true, it will be interesting to understand how cells determine which CENP-B boxes are methylated. One possibility is that in addition to CENP-A nucleosome-dependent epigenetic mechanisms, CENP-B box methylation could serve as a guide to identify the active HOR and resolve conflicts between HORs. Alternatively, transcription has been suggested to be required for CENP-A maintenance (Chen et al., 2015; Grenfell et al., 2016; Bobkov et al., 2018; Zhu et al., 2018), therefore it is possible that low CpG methylation at CDRs could regulate CENP-A deposition possibly through the differential recruitment of transcription factors.

### Future Directions

With the completion of the human genome, investigations of the organization, inheritance, and maintenance of CENP-A chromatin at a single chromosome level is more within our reach than ever. The recent telomere-to-telomere genome assembly has identified unique markers within the highly homogenous HORs (Altemose et al., 2022a; Nurk et al., 2022). Most unique markers fall within 10 kb of one another thus long sequencing reads generated through Oxford Nanopore or Pacbio sequencing can unambiguously map DNA sequences to the centromere. The next challenge is to identify CENP-A positions on these DNA sequences. A newly developed technique, DiMeLo-seq relies on antibody-directed localization of an exogenous adenine DNA methyltransferase Hia5 to CENP-A followed by long read sequencing to simultaneously read out DNA sequence and adenine methylation (Altemose et al., 2022b). Using DiMeLo-seq one can now map CENP-A nucleosomes confidently onto homogeneously repetitive DNA sequences. Challenges of low centromeric coverage due to the low representation of centromere sequences in the genome can be overcome by restriction enzyme based digestion of non-centromeric DNA sequences and selective sequencing of centromeric DNA (Gamba et al., 2021; Altemose et al., 2022b). The next frontier in understanding centromere inheritance and maintenance at the single molecule level is to be able to track CENP-A position using DiMeLo-seq or other approaches before and after CENP-A deposition (in G1-phase) and before and after CENP-A distribution (in S-phase) on the same chromatin fiber. Combining exogenous adenine methylation with exogenous GpC methylation (such as in NanoNOME (Lee et al., 2020)) or another DNA modification to perform pulse-chase experiments would enable such investigations. Currently, the validated human genome exists only for the model cell line Chm13. The complete sequences of centromeres in different cell types will enable detailed understanding of how centromere replication and maintenance are controlled. This provides a new opportunity to probe the mechanisms through which centromere assembly factors and centromeric DNA ensure stable centromere inheritance.
